# Inflammatory-hemodynamic interplay in severe intracranial stenosis: hs-CRP, vertebral artery flow, and posterior circulation infarction

**DOI:** 10.3389/fneur.2026.1829231

**Published:** 2026-08-25

**Authors:** Xiaofeng Yuan, Yan Zhang, Wenjing Ma, Yakun Ren, Jun Shao

**Affiliations:** 1 Kunshan Clinical Innovation Research Center of Nantong University, Kunshan, Jiangsu, China; 2 Department of Stroke Center, The First People's Hospital of Kunshan, Kunshan, Jiangsu, China; 3 Department of Ultrasound, The First People's Hospital of Kunshan, Kunshan, Jiangsu, China; 4 Department of Neurology, The First People's Hospital of Kunshan, Kunshan, Jiangsu, China

**Keywords:** hemodynamics, high-sensitivity C-reactive protein, intracranial atherosclerosis, mediation analysis, posterior circulation infarction

## Abstract

**Background:**

While systemic inflammation (hs-CRP) is linked to intracranial atherosclerosis, how it precisely translates into focal posterior circulation infarction (PCI) remains elusive. Previous studies largely treat inflammation and hemodynamics as isolated risk factors, leaving their potential synergistic mechanisms unquantified. We hypothesized that vertebral artery volume flow (VAVF) may partly account for the observed association between hs-CRP and PCI, thereby exploring a potential inflammatory-hemodynamic interaction in severe intracranial vertebral artery stenosis (IVAS).

**Methods:**

This retrospective cohort study included 253 consecutive patients with newly diagnosed severe IVAS. We assessed baseline hs-CRP, VAVF, and incident PCI using generalized additive models, exploratory mediation analysis, and network analyses.

**Results:**

During follow-up, 96 patients (37.9%) developed PCI. Each 1-mg/L increase in hs-CRP was associated with higher odds of PCI (adjusted OR, 1.26; 95% CI, 1.15–1.38; *p* < 0.001). The association was nonlinear, with an inflection point at 5.67 mg/L and a plateau above 13.28 mg/L. Network analysis identified hs-CRP as a central node within the biomarker network. Exploratory mediation analysis suggested that VAVF partly accounted for the association, with an estimated proportion mediated of 13.2% (*p* = 0.002). The association was strongest in the lowest VAVF tertile.

**Conclusion:**

Our findings support a potential inflammatory-hemodynamic interaction where systemic inflammation is associated with impaired local perfusion and increased risk of PCI. Identification of this “low-flow, high-inflammation” phenotype may provide a framework for future risk stratification and prospective evaluation of inflammation-targeted strategies.

## Introduction

Posterior circulation infarction (PCI) accounts for approximately 20–25% of all ischemic strokes and is associated with disproportionately higher mortality and disability rates compared to anterior circulation events (1–3). Intracranial vertebral artery stenosis (IVAS) represents a predominant etiology of PCI, particularly in Asian populations (4). Despite aggressive medical therapy, patients with symptomatic intracranial stenosis remain at substantial risk of recurrent stroke. Trials including WASID, SAMMPRIS, VISSIT, and CASSISS established guideline-directed medical therapy as first-line treatment, while revascularization is generally reserved for selected patients with recurrent ischemia or hemodynamic compromise (5–10). However, luminal stenosis alone may not fully capture the mechanistic and prognostic heterogeneity of intracranial atherosclerotic disease (ICAD). Recent evidence has emphasized that hemodynamic insufficiency, collateral status, infarction patterns, plaque characteristics, and embolic potential may provide complementary information for recurrent-stroke risk stratification and future trial design (11).

Recent paradigms in vascular biology and neuroimmunology have identified “residual inflammatory risk” (RIR) as a critical determinant of adverse cardiovascular outcomes (12). The 2025 Scientific Statement from the American College of Cardiology (ACC) highlighted high-sensitivity C-reactive protein (hs-CRP) as a cornerstone biomarker for risk stratification, independent of low-density lipoprotein cholesterol (LDL-C) levels (13). Crucially, hs-CRP is a canonical downstream biomarker of the interleukin-6 (IL-6) signaling pathway, reflecting the systemic activation of innate immunity (14). Elevated hs-CRP is not merely a passive bystander but a potential contributor in atherothrombosis, promoting plaque vulnerability and coagulation cascade activation (15, 16). As recently elucidated by Libby et al., inflammation is now recognized as an important contributor of atherosclerotic progression and endothelial instability, marking a fundamental shift from the traditional lipid-centric view (17). However, in the context of IVAS, the specific mechanisms by which systemic inflammation translates into focal ischemic events in the posterior circulation remain incompletely understood.

Hemodynamic compromise, specifically distal hypoperfusion caused by severe stenosis, is another established mechanism of PCI. The VERiTAS study highlighted that patients with compromised distal flow face a significantly higher risk of stroke recurrence (18). While hemodynamic compromise is a recognized precipitant of PCI, emerging evidence highlights the critical role of innate immunity in exacerbating this local vascular injury. Chronic inflammation can directly induce endothelial dysfunction and impair nitric oxide bioavailability, thereby compromising the autoregulatory capacity of distal cerebral vessels (19). Furthermore, the interplay between innate immunity and coagulation—termed thrombo-inflammation—may aggravate microvascular perfusion deficits (20). We hypothesize that systemic inflammation does not act in isolation but may partially exert its pro-atherogenic effects by aggravating hemodynamic insufficiency in the stenotic vertebral artery, creating a synergistic “double hit” of vulnerable plaque and low fluid shear stress (21). Current paradigms suggest that systemic low-grade inflammation may synergize with local hemodynamic alterations to precipitate ischemic events; however, this “immunothrombotic” cross-talk remains largely unquantified in patients with severe IVAS. Prior prospective data from the VERiTAS cohort showed that vertebrobasilar hypoperfusion independently amplifies recurrent stroke risk in posterior circulation ICAD, yet prior studies rarely integrated systemic inflammatory biomarkers with quantitative vascular flow metrics to evaluate their joint associations (18). To date, no studies have quantitatively investigated whether hemodynamic compromise may represent a potential pathway linking inflammation with PCI risk.

Therefore, this study aims to examine the associations among baseline hs-CRP, VAVF, and incident PCI in patients with severe IVAS. We further explored whether VAVF might statistically account for part of the association between hs-CRP and PCI and characterized the interrelationships among thrombo-inflammatory biomarkers.

## Materials and methods

### Study design and participants

We conducted a single-center, retrospective cohort study at The First People’s Hospital of Kunshan. Consecutive adult patients with newly diagnosed severe IVAS admitted to the Department of Neurology or Neurosurgery were screened for eligibility between January 2021 and December 2024. Eligible patients were followed from hospital discharge through the study cutoff date in July 2025. The study adhered to the principles of the Declaration of Helsinki. The Institutional Ethics Committee approved the study protocol (Approval No.2025–03-048-H00-K01) and waived the requirement for written informed consent due to the retrospective nature of the analysis. The reporting of this study conforms to the Strengthening the Reporting of Observational Studies in Epidemiology (STROBE) guidelines (22).

### Inclusion and exclusion criteria

We included patients satisfying the following criteria: (1) newly diagnosed severe IVAS (≥70% stenosis) confirmed by computed tomography angiography (CTA) or digital subtraction angiography (DSA) utilizing the North American Symptomatic Carotid Endarterectomy Trial (NASCET) methodology; (2) absence of any prior or acute PCI on baseline neuroimaging, confirmed by negative findings on both Diffusion-Weighted Imaging (DWI) and structural MRI sequences (T1/T2/fluid-attenuated inversion recovery [FLAIR]); (3) availability of complete baseline data, specifically fasting hs-CRP levels and vertebral artery hemodynamic parameters assessed within 72 h of admission; and (4) prescription of guideline-directed medical therapy (GDMT), comprising standard antiplatelet and statin regimens, at discharge.

We excluded patients meeting the following criteria: (1) non-atherosclerotic arterial stenosis (e.g., dissection, arteritis, or Moyamoya disease); (2) potential cardioembolic sources (e.g., atrial fibrillation or valvular heart disease); (3) concurrent severe stenosis (≥70%) or occlusion involving the anterior circulation, basilar artery, or proximal vessels (e.g., subclavian or extracranial vertebral artery), to isolate the hemodynamic impact of the intracranial lesion; (4) active infection, malignancy, or systemic immune disorders at baseline that could non-specifically elevate inflammatory markers; or (5) receipt of endovascular stenting or surgical bypass during the follow-up period.

### Data collection and definitions

Demographic data, vascular risk factors, and clinical measurements were comprehensively collected at baseline. Vascular risk factors were defined using standard criteria: hypertension as systolic blood pressure ≥140 mmHg, diastolic blood pressure ≥90 mmHg, or antihypertensive use; diabetes mellitus as fasting glucose ≥7.0 mmol/L, glycated hemoglobin (HbA1c) ≥ 6.5%, or antidiabetic treatment; dyslipidemia as total cholesterol ≥5.2 mmol/L, LDL-C ≥ 3.4 mmol/L, or lipid-lowering therapy; and smoking status as tobacco use within the past 6 months. Fasting venous blood samples were obtained within 72 h of admission. Thrombo-inflammatory biomarkers (hs-CRP, fibrinogen, and D-dimer) and lipid profiles were measured using standard biochemical and coagulation assays. Serum hs-CRP was measured using the MULTIGENT CRP Vario latex immunoturbidimetric assay on an ARCHITECT c8000 analyzer (Abbott Laboratories; assay range, 0.1–160 mg/L).

VAVF was measured at the intervertebral V2 segment using an EPIQ CVx color Doppler ultrasound system (Philips, Best, The Netherlands) with a 3–12-MHz linear-array transducer. VAD and TAPV were measured three times by one of three certified vascular sonographers with at least 5 years of experience, and the mean values were used for analysis. VAVF was calculated as $Q=(\text{TAPV}÷2)\times [\pi \times {\left(\mathrm{VAD}÷2\right)}^{2}]\times 60$ (23). Interobserver reproducibility was assessed in 10 patients by two independent sonographers using a two-way random-effects, absolute-agreement ICC. For unilateral occlusion proximal to the posterior inferior cerebellar artery (PICA), VAVF was the flow volume of the contralateral vertebral artery; and for unilateral occlusion distal to the PICA or severe stenosis, VAVF was calculated as the sum of flow volumes from both vertebral arteries. These assessments were performed by experienced sonographers who were inherently blinded to the patients’ subsequent clinical course.

### Outcome assessment

The primary outcome was incident PCI, defined as acute focal neurological deficits in the vertebrobasilar territory accompanied by a new ischemic lesion on DWI or T2-weighted MRI. Outcomes were ascertained through institutional electronic medical records and structured telephone interviews. Telephone-ascertained events were classified as PCI when the patient or proxy reported hospitalization and a physician diagnosis of MRI-confirmed posterior circulation infarction. Follow-up extended from discharge to the first PCI event, last contact, or July 31, 2025. Event dates were considered available when documented in institutional records or specifically reported during telephone follow-up. Available hospitalization and neuroimaging records were reviewed to verify suspected events.

### Statistical analysis

Data distribution was initially assessed using the Shapiro–Wilk test. Continuous variables are expressed as mean ± standard deviation (SD) or median (interquartile range [IQR]) and were compared using Student’s *t*-test or the Mann–Whitney *U* test, respectively. Categorical data are presented as frequencies (*n*, %) and were analyzed using the Pearson *χ*^2^ test or Fisher’s exact test, as appropriate.

Core study variables were complete for all enrolled patients. Missing values were only present for BMI in 12 participants. We performed multiple imputation based on fully conditional specification with 5 imputed datasets to handle the missing BMI data, and pooled the results across all imputed datasets for the final analysis.

To evaluate the independent association between hs-CRP and PCI, a multivariable logistic regression model was constructed adjusting for age, sex, clinically relevant thrombo-inflammatory biomarkers, and covariates with univariate *p* < 0.10 and a variance inflation factor (VIF) < 5. Variable selection was additionally guided by clinical relevance and previously reported associations with stroke risk. Multivariable logistic regression was selected over Cox proportional hazards modeling to derive odds ratios (ORs), primarily due to the unavailability of precise PCI onset dates for a subset of patients identified via telephone follow-up. Logistic regression was selected as the primary analysis because precise onset dates were unavailable for 63 PCI events, precluding complete time-to-event analysis without imputing event times. The discrimination performance of the final multivariable model was evaluated using the C-statistic, and calibration was assessed using the Hosmer–Lemeshow test. As sensitivity analyses, Cox proportional hazards regression and restricted logistic regression were performed among 190 patients with evaluable time-to-event data, including 33 patients with available PCI onset dates and 157 patients who remained event-free. Model I and Model II were specified consistently with the primary analysis. Given the limited number of PCI events with evaluable onset date, Model III was restricted to age, sex, LDL-C, VAVF, and statin therapy to limit model complexity and reduce the risk of overfitting. The proportional hazards assumption was assessed using Schoenfeld residuals.

Generalized Additive Models (GAM) with penalized spline smoothing were used to characterize the potential non-linear dose–response relationship between hs-CRP and PCI. The model was adjusted for potential confounders, including age, sex, high-density lipoprotein cholesterol (HDL-C), LDL-C, fibrinogen, D-dimer, lipoprotein-associated phospholipase A2 (Lp-PLA2), lipoprotein(a) [Lp(a)], VAVF, antiplatelet therapy and statin therapy. A piecewise linear regression model incorporating a recursive algorithm was employed to identify two distinct inflection points, representing the risk threshold and the saturation point, respectively, with model superiority determined via the log-likelihood ratio test. Furthermore, stratified analyses were performed across prespecified subgroups, where the statistical significance of interaction terms was evaluated using the likelihood ratio test.

Exploratory mediation analysis was conducted within the potential outcomes framework to estimate the overall, indirect, and direct components of the association between hs-CRP and PCI. A linear regression model was specified for the mediator VAVF alongside a generalized linear model with a probit link function for the outcome PCI. Statistical significance and 95% confidence intervals (CIs) were estimated via nonparametric bootstrapping with 5,000 resamples.

A partial correlation network was constructed to map the associations among thrombo-inflammatory biomarkers. To isolate the unique association between each pair of biomarkers, partial Spearman rank correlation coefficients were calculated adjusting for potential confounders, including age, sex, lipid profiles, and medication history, as well as all other biomarkers within the network. Edges in the network represented statistically significant partial correlations (*p* < 0.05). Finally, network topology was characterized using centrality indices, including strength, betweenness, and closeness. All statistical analyses were conducted using R software (http://www.R-project.org, The R Foundation), Empower Stats (http://www.empowerstats.com, X&Y Solutions, Inc., Boston, MA), and IBM SPSS Statistics 26.0, with network visualization performed via Cytoscape version 3.9.1. A two-sided *p* < 0.05 indicated statistical significance.

## Results

### Study population and baseline characteristics

**Figure 1:**
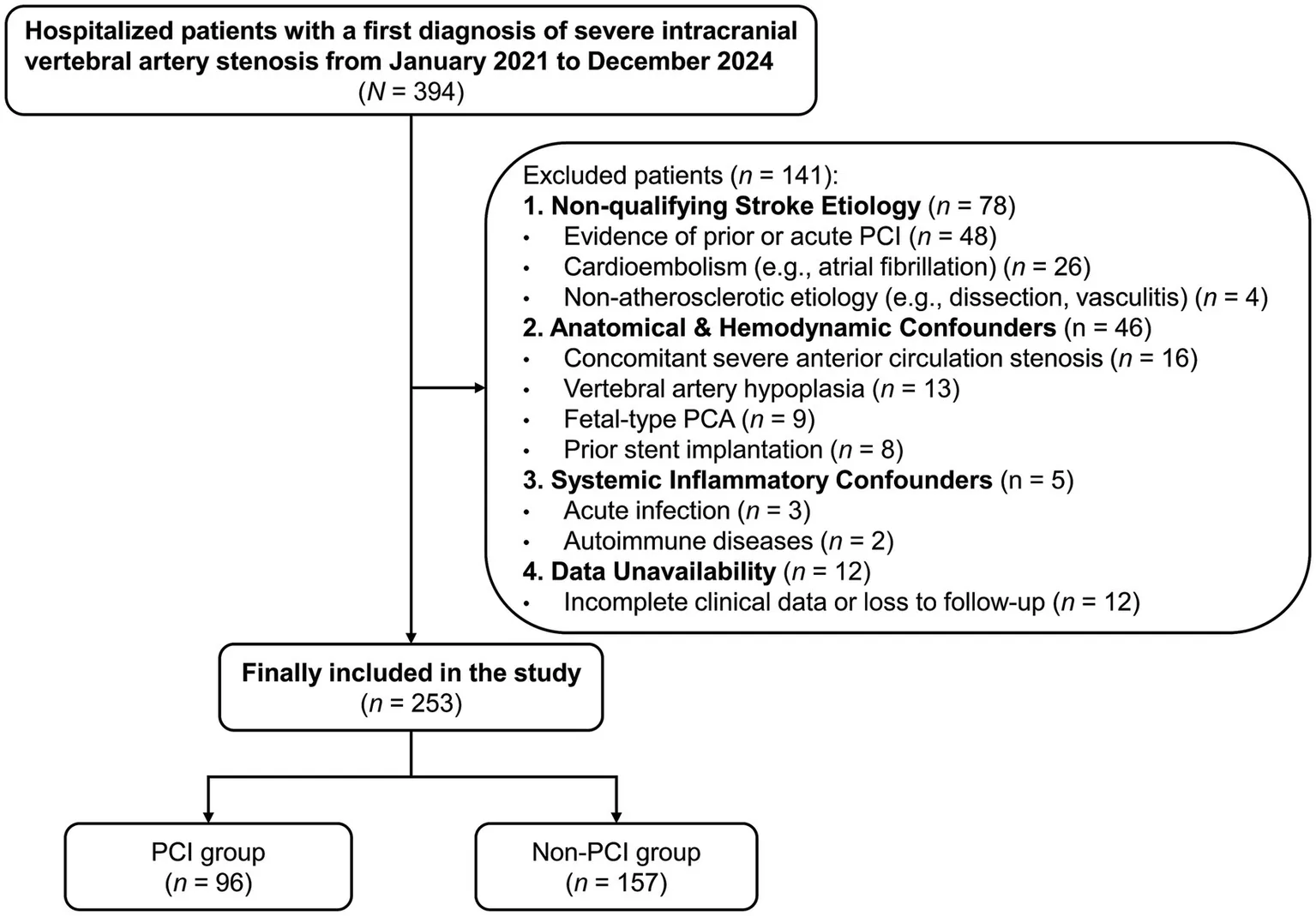
Flowchart of patient recruitment and study design. PCA, posterior cerebral artery; PCI, posterior circulation infarction.

Of the 394 patients screened, 253 met the inclusion criteria and were included in the final analysis (Figure 1). Among 253 patients, 96 (37.9%) developed PCI and 157 (62.1%) remained event-free. Median follow-up was 28 months (IQR, 22–32; range, 10–45). Of the 96 events, 21 were identified from institutional records and 75 through telephone follow-up. Onset dates were available for 33 events—21 from institutional records and 12 from patient or proxy reports—and unavailable for 63 events (65.6% of PCI events; 24.9% of the cohort). Among the 33 events with evaluable timing, median time to PCI was 18 months (IQR, 15–22; range, 12–37).

Baseline demographics and vascular risk factors, including age, sex, body mass index (BMI), hypertension, diabetes mellitus, and smoking status, did not differ significantly between groups. However, the PCI group exhibited significantly higher median hs-CRP (11.5 vs. 3.8 mg/L; *p* < 0.001), fibrinogen, D-dimer, and LDL-C levels (all *p* < 0.001), alongside lower HDL-C (*p* = 0.025). VAVF was significantly lower in the PCI group (111.9 vs. 155.9 mL/min; *p* < 0.001). Additionally, pre-admission use of antiplatelets (*p* = 0.004) and statins (*p* < 0.001) was less frequent in the PCI group (Table 1). Interobserver agreement was excellent for both VAD and TAPV, with ICCs of 0.933 (95% CI, 0.738–0.997) and 0.937 (95% CI, 0.859–0.990), respectively.

**Table 1:** Baseline characteristics of patients stratified by the occurrence of PCI.

Variable	Total (*N* = 253)	Non-PCI (*n* = 157)	PCI (*n* = 96)	*P-*value
Demographics				
Age, years	66.4 ± 11.6	67.0 ± 11.3	65.4 ± 12.1	0.277
Male sex, *n* (%)	185 (73.1%)	111 (70.7%)	74 (77.1%)	0.266
BMI, kg/m^2^	25.0 ± 3.2	24.7 ± 3.3	25.3 ± 3.1	0.168
Vascular Risk Factors, *n* (%)				
Hypertension	198 (78.3%)	119 (75.8%)	79 (82.3%)	0.224
Diabetes mellitus	111 (43.9%)	71 (45.2%)	40 (41.7%)	0.580
Dyslipidemia	153 (60.5%)	95 (60.5%)	58 (60.4%)	0.988
Coronary heart disease	32 (12.6%)	24 (15.3%)	8 (8.3%)	0.106
Smoking status	79 (31.2%)	45 (28.7%)	34 (35.4%)	0.261
SBP, mmHg	142.3 ± 19.4	140.8 ± 18.9	144.6 ± 20.0	0.129
DBP, mmHg	83.1 ± 12.2	83.3 ± 12.7	82.9 ± 11.3	0.790
Laboratory Findings				
Fasting glucose, mmol/L	6.6 ± 2.4	6.6 ± 2.5	6.5 ± 2.2	0.743
HbA1c, %	7.0 ± 1.7	6.9 ± 1.7	7.1 ± 1.6	0.386
Total cholesterol, mmol/L	4.3 ± 1.0	4.3 ± 1.0	4.5 ± 1.0	0.134
LDL-C, mmol/L	2.6 ± 1.2	2.3 ± 1.1	3.0 ± 1.2	**<0.001**
HDL-C, mmol/L	1.1 ± 0.3	1.1 ± 0.3	1.0 ± 0.3	**0.025**
Triglycerides, mmol/L	1.6 ± 0.9	1.6 ± 0.9	1.7 ± 0.9	0.775
Homocysteine, μmol/L	12.6 ± 6.3	12.7 ± 7.0	12.4 ± 4.9	0.783
Lp(a), mg/L	175.2 (119.6–243.5)	172.1 (116.8–242.8)	189.7 (135.7–252.3)	0.200
Lp-PLA2, ng/mL	183.6 (138.0–223.5)	170.7 (141.2–212.4)	196.4 (133.4–243.9)	0.054
Inflammatory and Coagulation Markers				
hs-CRP, mg/L	5.2 (2.5–9.5)	3.8 (1.9–5.9)	11.5 (5.4–15.0)	**<0.001**
Fibrinogen, g/L	3.1 (2.4–3.9)	2.9 (2.3–3.6)	3.6 (2.8–4.2)	**<0.001**
D-dimer, mg/L	0.8 (0.3–1.4)	0.7 (0.2–1.2)	1.1 (0.5–1.9)	**<0.001**
Hemodynamic Parameter				
VAVF, mL/min	139.1 ± 52.2	155.9 ± 52.6	111.9 ± 38.4	**<0.001**
Medication Prior to Admission, *n* (%)				
Antiplatelet therapy	77 (30.3%)	58 (36.9%)	19 (19.8%)	**0.004**
Statin therapy	88 (34.8%)	72 (45.9%)	16 (16.7%)	**<0.001**

BMI, body mass index; DBP, diastolic blood pressure; HbA1c, glycosylated hemoglobin; HDL-C, high-density lipoprotein cholesterol; hs-CRP, high-sensitivity C-reactive protein; IQR, interquartile range; LDL-C, low-density lipoprotein cholesterol; Lp(a), lipoprotein(a); Lp-PLA2, lipoprotein-associated phospholipase A2; PCI, posterior circulation infarction; SBP, systolic blood pressure; SD, standard deviation; VAVF, vertebral artery volume flow. Data are presented as mean ± SD, median (IQR), or number (percentage). *P*-values were calculated using Student’s *t*-test, Mann–Whitney *U* test, or Pearson *χ*^2^ test, as appropriate. Bold values indicate statistical significance (*P* < 0.05).

### Association between baseline hs-CRP and PCI

In univariate analysis, baseline hs-CRP was associated with PCI (OR, 1.33; 95% CI, 1.24–1.44; *p* < 0.001) (Supplementary Table S1). The association remained significant after multivariable adjustment (Table 2). Each 1-mg/L increase in hs-CRP was associated with 26% higher odds of PCI (OR, 1.26; 95% CI, 1.15–1.38; *p* < 0.001). Patients in the highest hs-CRP tertile had more than 10-fold higher odds of PCI than those in the lowest tertile (OR, 10.18; 95% CI, 3.79–27.40; *p* < 0.001). The final multivariable model showed good discrimination (C-index = 0.863; 95% CI, 0.814–0.911) and satisfactory calibration (Hosmer–Lemeshow test: *p* = 0.174). In sensitivity analyses restricted to patients with evaluable time-to-event data, higher baseline hs-CRP remained associated with PCI in Model III of the Cox analysis (HR, 1.37; 95% CI, 1.24–1.53; *p* < 0.001) and restricted logistic regression model (OR, 1.56; 95% CI, 1.31–1.87; *p* < 0.001) (Supplementary Table S2). No evidence of violation of the proportional hazards assumption was observed in the Cox models (all Schoenfeld *p* > 0.05).

**Table 2:** Association between baseline hs-CRP and PCI in different adjusted models.

Variable	Model I		Model II		Model III	
	OR (95% CI)	*P*	OR (95% CI)	*P*	OR (95% CI)	*P*
hs-CRP (per 1 mg/L)	1.33 (1.24–1.44)	<0.001	1.33 (1.24–1.44)	<0.001	1.26 (1.15–1.38)	<0.001
hs-CRP Tertiles (mg/L)						
T1 (≤3.43 mg/L)	Reference		Reference		Reference	
T2 (>3.43, ≤7.31 mg/L)	1.64 (0.73–3.65)	0.229	1.64 (0.73–3.68)	0.234	1.15 (0.45–2.91)	0.772
T3 (>7.31 mg/L)	20.84 (9.40–46.21)	<0.001	20.59 (9.28–45.68)	<0.001	10.18 (3.79–27.40)	<0.001
*P* for trend	<0.001		<0.001		<0.001	

CI, confidence interval; hs-CRP, high-sensitivity C-reactive protein; OR, odds ratio. Note: Model I was unadjusted. Model II was adjusted for age and sex. Model III was adjusted for age, sex, HDL-C, LDL-C, fibrinogen, D-dimer, Lp-PLA2, Lp(a), VAVF, antiplatelet therapy, and statin therapy.

### Threshold effect and saturation analysis

**Figure 2:**
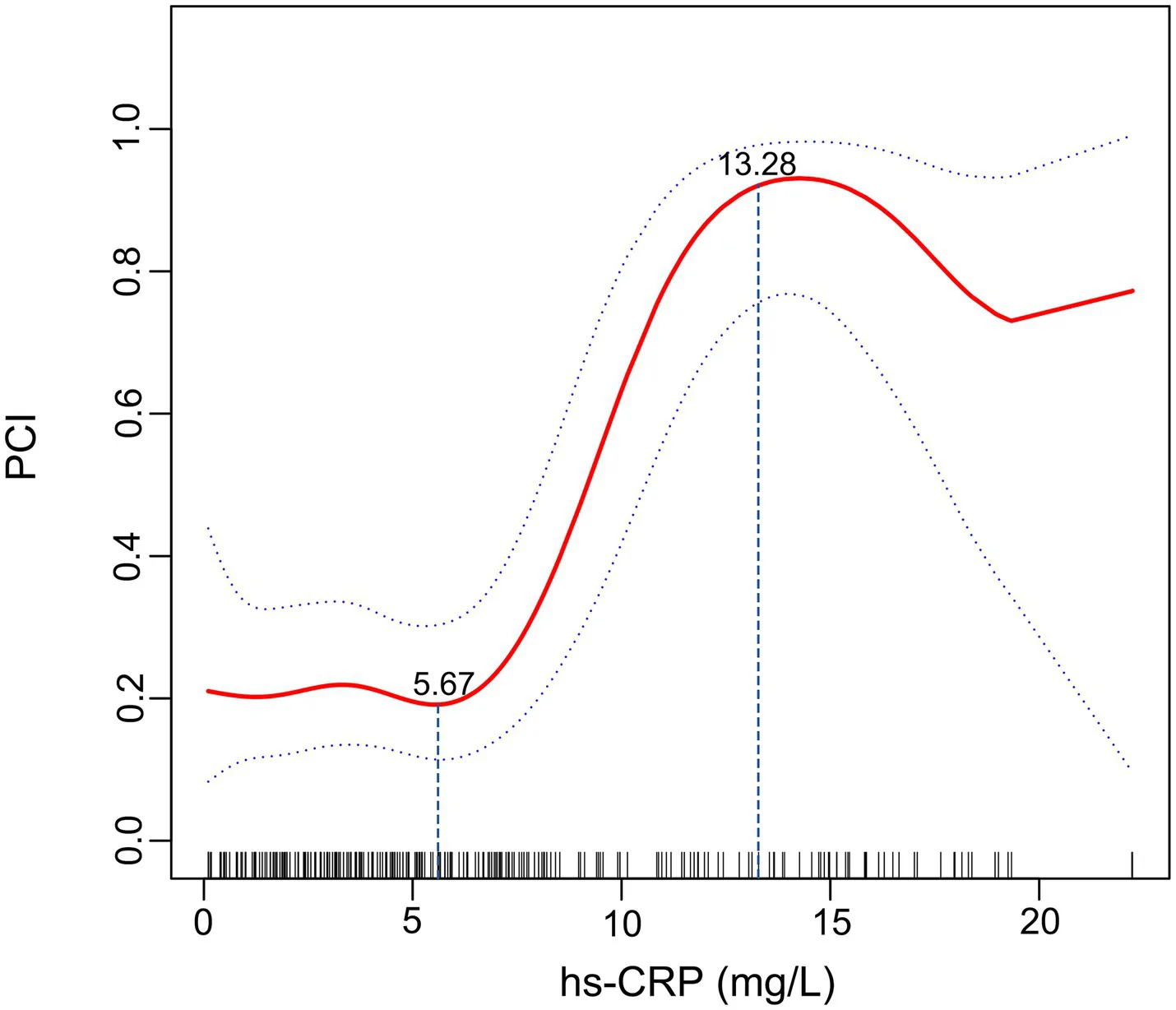
Non-linear dose–response relationship between baseline hs-CRP levels and the risk of PCI. The model was adjusted for age, sex, HDL-C, LDL-C, fibrinogen, D-dimer, Lp-PLA2, Lp(a), VAVF, antiplatelet therapy, and statin therapy. The red solid line represents the smooth curve fit, with the blue dashed lines representing the 95% CI. Vertical dashed lines indicate the inflection points at 5.67 mg/L and 13.28 mg/L. The bottom rug plot shows the distribution of hs-CRP measurements. HDL-C, high-density lipoprotein cholesterol; hs-CRP, high-sensitivity C-reactive protein; LDL-C, low-density lipoprotein cholesterol; Lp(a), lipoprotein(a); Lp-PLA2, lipoprotein-associated phospholipase A2; PCI, posterior circulation infarction; VAVF, vertebral artery volume flow.

After adjusting for the aforementioned confounders, the dose–response analysis revealed a non-linear association between hs-CRP and PCI (Figure 2). Threshold effect analysis identified two distinct inflection points at 5.67 mg/L and 13.28 mg/L. Below 5.67 mg/L, no significant association was observed. Between 5.67 and 13.28 mg/L, the odds of PCI increased with hs-CRP (OR, 1.45 per mg/L; *p* < 0.001), followed by a plateau above 13.28 mg/L.

### Subgroup analyses and interaction effects

**Figure 3:**
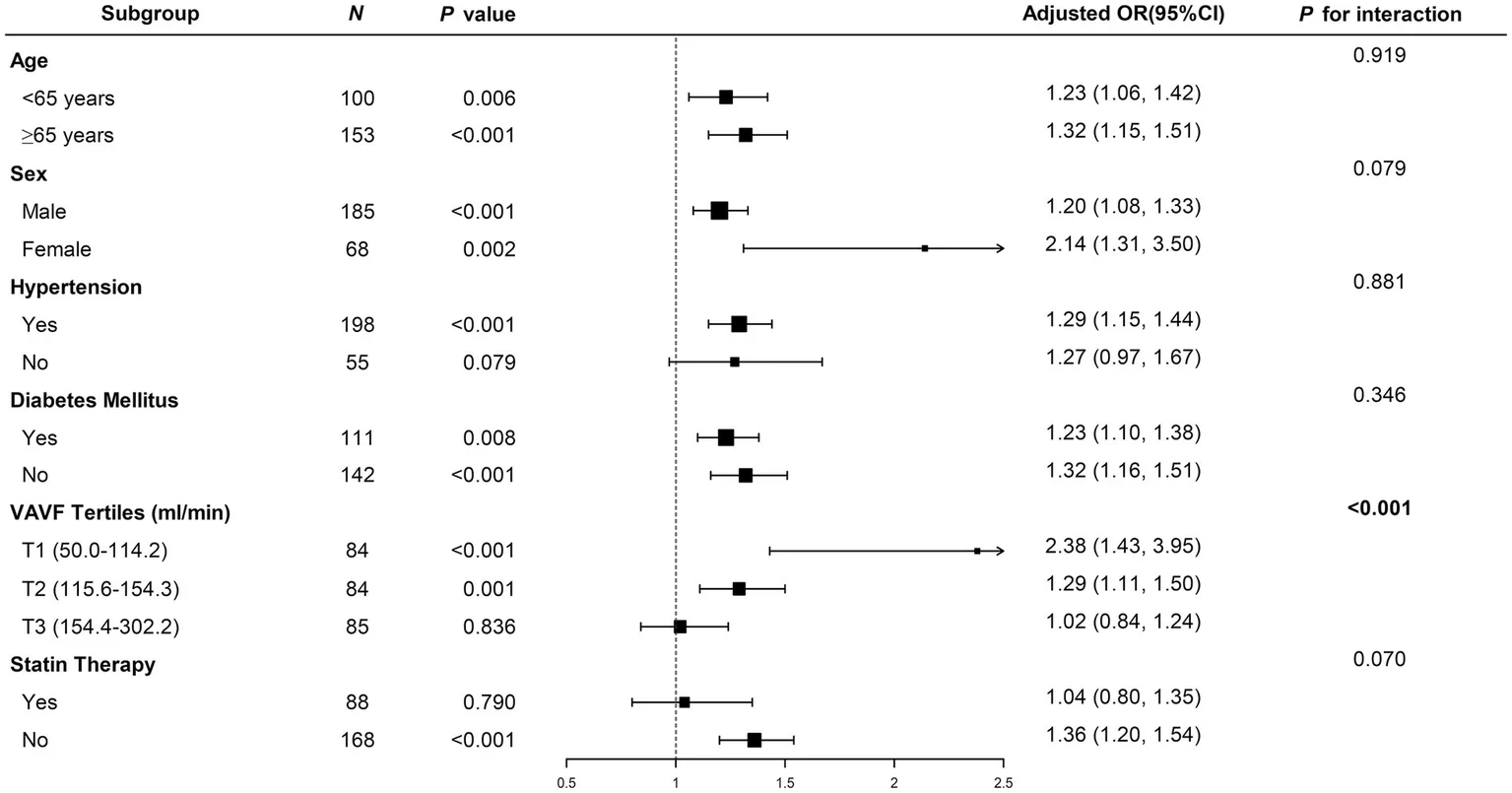
Forest plot of subgroup analyses for the association between hs-CRP and PCI. Black squares represent the adjusted odds ratios (ORs), with the size of each square proportional to the statistical weight of each subgroup. Horizontal lines indicate the 95% CIs. Multivariable models were adjusted for age, sex, HDL-C, LDL-C, fibrinogen, D-dimer, Lp-PLA2, Lp(a), antiplatelet therapy, and statin therapy, excluding the stratification variable in each subgroup. The *p*-values for interaction are displayed in the rightmost column. CI, confidence interval; hs-CRP, high-sensitivity C-reactive protein; LDL-C, low-density lipoprotein cholesterol; Lp(a), lipoprotein(a); Lp-PLA2, lipoprotein-associated phospholipase A2; OR, odds ratio; PCI, posterior circulation infarction; VAVF, vertebral artery volume flow.

The association between hs-CRP and PCI remained consistent across age, sex, hypertension, and diabetes subgroups (*P* for interaction > 0.05) (Figure 3). However, hemodynamic status significantly modified this risk (*P* for interaction < 0.001): the association was strongest in the lowest VAVF tertile (OR, 2.38; 95% CI, 1.43–3.95) but was not statistically significant in the highest tertile (OR, 1.02; 95% CI, 0.84–1.24). Additionally, a marginal interaction (*p* = 0.070) was observed regarding statin therapy: the association was significant in non-users (OR, 1.36; 95% CI, 1.20–1.54) but absent in users (OR, 1.04; 95% CI, 0.80–1.35).

### Exploratory mediation analysis of hemodynamics

**Figure 4:**
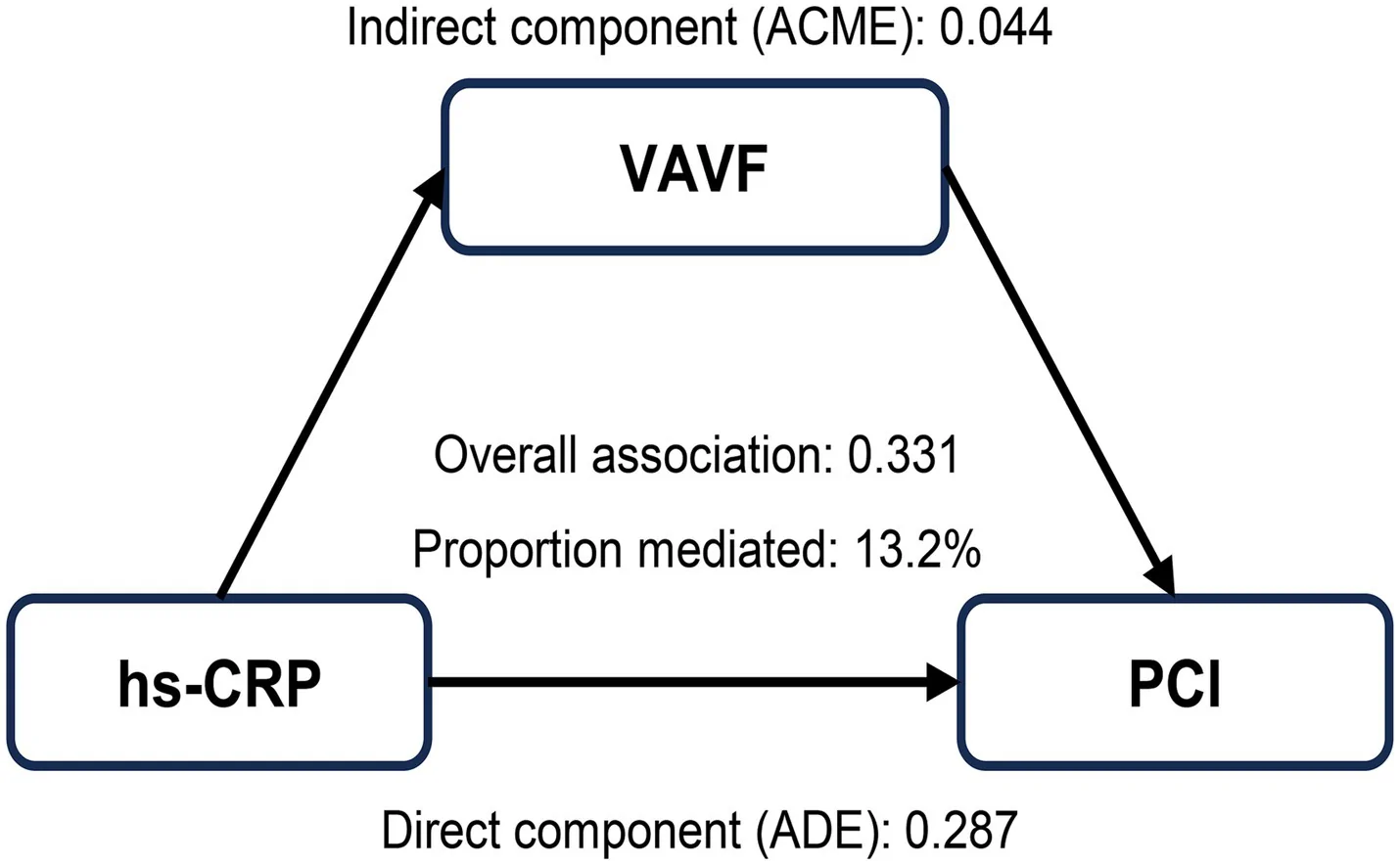
Path diagram of the exploratory mediation analysis evaluating VAVF as a potential mediator of the association between hs-CRP and PCI. The estimated indirect component (ACME) was 0.044 (95% *CI*, 0.011–0.082; *p* = 0.002), the direct component (ADE) was 0.287 (95% *CI*, 0.179–0.405; *p* < 0.001), and the overall association was 0.331 (*p* < 0.001); the estimated proportion mediated was 13.2%. Models were adjusted for prespecified covariates as described in the Methods. Arrows indicate the hypothesized analytical pathway and do not establish causality. ACME, average causal mediation effect; ADE, average direct effect; hs-CRP, high-sensitivity C-reactive protein; PCI, posterior circulation infarction; VAVF, vertebral artery volume flow.

Exploratory mediation analysis was performed to evaluate whether VAVF might partly account for the association between hs-CRP and PCI (Figure 4). The estimated overall association between hs-CRP and PCI was statistically significant (*β* = 0.331; *p* < 0.001). The estimated indirect component through VAVF was 0.044 (95% CI, 0.011–0.082; *p* = 0.002), corresponding to an estimated proportion mediated of 13.2%. The estimated direct component remained statistically significant (*β* = 0.287; 95% CI, 0.179–0.405; *p* < 0.001).

### Network analysis of inflammatory and thrombotic biomarkers

**Figure 5:**
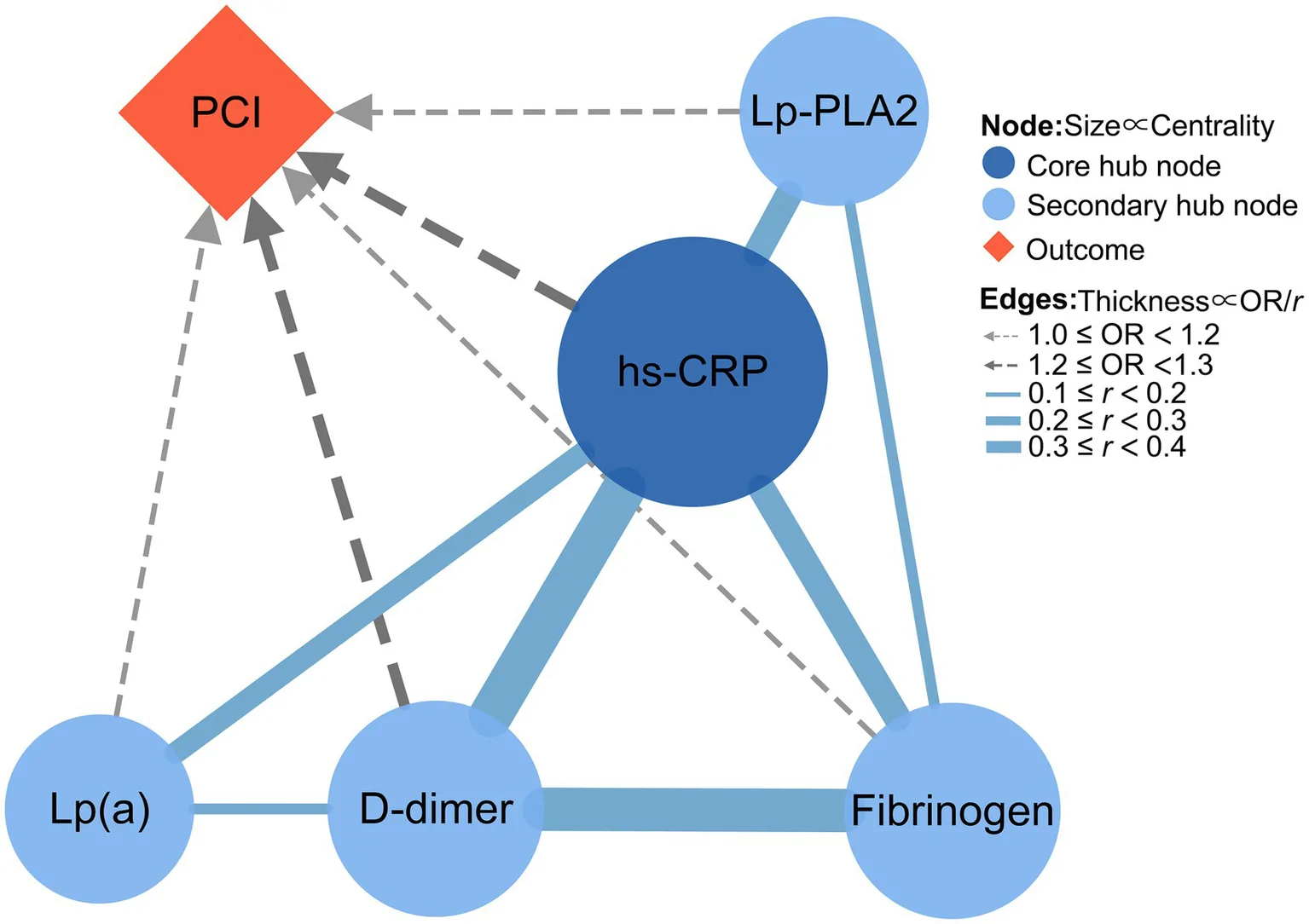
Network analysis of inflammatory and thrombotic biomarkers and PCI. Node size is proportional to strength centrality. Undirected edges represent significant partial correlations (*p* < 0.05), while directed arrows indicate the adjusted ORs for the association between biomarkers and PCI. Analyses were adjusted for age, sex, HDL-C, LDL-C, VAVF, antiplatelet therapy, statin therapy, and concurrent biomarkers within the network. Edge thickness is proportional to the magnitude of the correlation coefficient (*r*) or OR. hs-CRP, high-sensitivity C-reactive protein; Lp(a), lipoprotein(a); Lp-PLA2, lipoprotein-associated phospholipase A2; OR, odds ratio; PCI, posterior circulation infarction; VAVF, vertebral artery volume flow.

Network analysis revealed a highly interconnected structure among the studied biomarkers and PCI, characterized by a network density of 0.800 and a clustering coefficient of 0.844 (Figure 5). Hs-CRP emerged as the central hub of this network, exhibiting the highest strength and betweenness centrality indices. Significant partial Spearman rank correlations linked hs-CRP to D-dimer (*r* = 0.306), Lp(a) (*r* = 0.259), Lp-PLA2 (*r* = 0.249), and fibrinogen (*r* = 0.221) (all *p* < 0.001). In the adjusted outcome model, hs-CRP remained associated with PCI (OR, 1.26; 95% CI, 1.15–1.38), whereas the other biomarkers were not significantly associated with PCI.

## Discussion

In this retrospective cohort study of patients with severe IVAS, we identified evidence supporting a potential “inflammatory-hemodynamic” interaction associated with PCI occurrence. While the prognostic value of hs-CRP is well-established in coronary artery disease and anterior circulation stroke (12, 15), our study extends this evidence by examining the potential contribution of hemodynamic impairment to the association between inflammation and PCI in the vertebrobasilar system, a vascular territory associated with higher mortality compared to the anterior circulation (24). Higher baseline hs-CRP was independently associated with PCI and showed a nonlinear dose–response relationship, with an inflection point at 5.67 mg/L. The association between hs-CRP and PCI was strongest among patients with the lowest VAVF, consistent with the “double-hit” hypothesis proposed in the Introduction (21). Furthermore, our exploratory mediation analysis suggested that hemodynamic impairment might partly account for the association between inflammation and PCI risk, with an estimated proportion mediated of 13.2%. This finding points to a possible biological link between systemic inflammation and impaired local perfusion, although the direction and temporal sequence of this relationship cannot be established from the present data (18). These findings align with the evolving approach to ICAD risk assessment that incorporates inflammatory, plaque, embolic, and hemodynamic characteristics in addition to anatomical stenosis (9–11).

The persistent risk of recurrent stroke despite the aggressive medical protocols established by the SAMMPRIS and VIST trials highlights the need to identify additional contributors to residual risk. In this context, our findings extend the framework of RIR to the posterior circulation and are consistent with the emphasis placed on inflammation in the 2025 ACC Scientific Statement (13). The significantly higher median baseline hs-CRP level in the PCI group suggests a greater systemic inflammatory burden among patients who subsequently developed PCI. This finding is consistent with previous evidence that the combined burden of inflammation and dyslipidemia is associated with increased stroke risk beyond lipid levels alone (25). Furthermore, recent high-resolution MRI findings have associated RIR with intracranial plaque vulnerability and enhancement (26). Compared with the carotid circulation, the posterior circulation may be more susceptible to lower shear stress and impaired microembolic washout (27), which could increase vulnerability to the combined effects of inflammatory and hemodynamic abnormalities and supports consideration of both lipid-related and inflammatory pathways (28).

To elucidate the potential biological mechanisms, our results suggest a possible biochemical-hemodynamic coupling. Biologically, C-reactive protein may participate in endothelial dysfunction and vascular inflammation through multiple biological pathways. Recent molecular reviews indicate that inflammation may downregulate endothelial nitric oxide synthase (eNOS) and promote endothelin-1 release (19), a mechanism supported by foundational *in vivo* studies (29). These effects could be superimposed on fixed anatomical stenosis and further compromise VAVF. Concurrently, severe stenosis creates a local environment of low fluid shear stress. According to a 2025 review in ATVB, this low-shear environment is associated with endothelial activation and increased expression of adhesion molecules (21), potentially contributing to lumen narrowing. Our network analysis identified hs-CRP as a central node within the inflammatory biomarker network associated with PCI risk. This finding is consistent with the concept of residual inflammatory risk, which persists despite optimal statin therapy (30). One potential explanation for this observed interaction is the immunothrombosis paradigm (31). Reduced VAVF and low shear stress may promote local endothelial activation, while an elevated systemic inflammatory burden may facilitate immune-cell recruitment and thrombo-inflammatory activity. Together, these processes could contribute to impaired perfusion, plaque instability, and ischemic events. However, these mechanisms represent biologically plausible interpretations and were not directly demonstrated in the present study.

Beyond the overall association, the plateau observed above 13.28 mg/L provides additional insight into the nonlinear relationship between hs-CRP and PCI. This pattern may reflect a reduced incremental association at higher inflammatory levels, consistent with nonlinear relationships reported in other stroke cohorts using generalized additive models (32). The observed increase in the odds of PCI above 5.67 mg/L may also provide a useful framework for identifying patients with a heightened inflammatory burden and informing future risk-stratification studies. However, this data-derived value should not yet be interpreted as a validated clinical threshold. The association between hs-CRP and PCI appeared attenuated among statin users, although the interaction did not reach statistical significance. This observation is compatible with the pleiotropic anti-inflammatory effects of statins, as reviewed in Molecular Medicine (33). These findings further suggest that patients with a combined “low-flow, high-inflammation” profile may represent a clinically relevant subgroup, highlighting the potential value of integrating inflammatory biomarkers with hemodynamic assessment in future individualized risk-stratification strategies. Such an approach is consistent with recent discussions of precision medicine targeting RIR (34). Therapeutically, targeting the IL-1β/IL-6/CRP inflammatory pathway represents a potential area of investigation for secondary stroke prevention. CANTOS demonstrated that IL-1β inhibition reduced cardiovascular events independently of lipid lowering (35). Low-dose colchicine reduced cardiovascular events in the LoDoCo2 trial (36) among patients with chronic coronary disease, whereas the CONVINCE trial (37) showed a non-significant reduction in recurrent vascular events following non-cardioembolic stroke. Ziltivekimab substantially reduced inflammatory biomarkers in the phase 2 RESCUE trial (38), while its effects on clinical cardiovascular outcomes are being evaluated in the ZEUS trial (39). Although these studies provide a rationale for investigating inflammation-targeted therapy in atherosclerotic disease, their applicability to patients with severe IVAS remains uncertain. Our findings raise the possibility that patients with the identified “low-flow, high-inflammation” phenotype may represent a candidate population for future evaluation of inflammation-targeted or hemodynamic strategies. These therapeutic implications are hypothesis-generating and require prospective validation in adequately powered randomized controlled trials before they can inform clinical practice.

The strengths of this study include the integration of systemic inflammatory biomarkers with quantitative vertebral artery flow measurements, assessment of nonlinear and subgroup relationships, and standardized ultrasound evaluation with excellent interobserver agreement. In addition, the consistency of the findings across the primary logistic regression and complementary Cox and restricted logistic sensitivity analyses strengthens the observed association between hs-CRP and PCI.

Our study has several limitations. First, the retrospective observational design and concurrent baseline measurement of hs-CRP and VAVF preclude definitive causal inference, because the temporal sequence between inflammation and hemodynamic impairment could not be established. Residual confounding cannot be excluded, and the assumptions required for mediation analysis cannot be fully verified. Therefore, the mediation findings should be interpreted as exploratory and hypothesis-generating. Second, precise onset dates were unavailable for 63 PCI events; therefore, logistic regression was used as the primary analysis. Although sensitivity analyses restricted to patients with evaluable time-to-event data yielded directionally consistent results, exclusion of events with unknown timing may have introduced selection bias, and the primary analysis could not fully account for variation in follow-up duration. Third, our assessments relied on single baseline measurements and indirect inferences. Specifically, plaque vulnerability was inferred from biomarkers rather than directly visualized via high-resolution vessel wall imaging (26), and VAVF measurements, while reliable, remain operator-dependent (18). Furthermore, recent evidence suggests that the longitudinal trajectory of inflammatory markers may offer superior prognostic value compared to a single baseline hs-CRP reading (40). Finally, this was a single-center study focusing specifically on severe IVAS, which may limit generalizability to other ICAD populations. Multicenter prospective studies incorporating standardized outcome verification, serial measurements, and multimodal imaging are warranted.

## Conclusion

In conclusion, elevated baseline hs-CRP was associated with impaired vertebral artery flow and a higher risk of PCI in patients with severe IVAS. Exploratory mediation findings suggested that hemodynamic compromise may partly account for this association. These findings are hypothesis-generating and warrant validation in prospective longitudinal studies.

## Data Availability

The raw data supporting the conclusions of this article will be made available by the authors, without undue reservation.
