# Supplementary material for: Inflammatory-hemodynamic interplay in severe intracranial stenosis: hs-CRP, vertebral artery flow, and posterior circulation infarction
Source: Front Neurol. 2026 Aug 25;17:1829231. doi: 10.3389/fneur.2026.1829231 (PMC13549819; doi:10.3389/fneur.2026.1829231)
Supplement: Supplementary file 1 [file Table_1.DOCX]

**Table S1.** Predictors of PCI by univariate analysis.

| **Variable** | **Statistics** | **OR（95%CI）** | ***P*-value** |
| --- | --- | --- | --- |
| **Demographics** | | | |
| Age, years | 66.42 ± 11.58 | 0.99 (0.97, 1.01) | 0.277 |
| Male sex, *n* (%) | 185 (73.12%) | 1.39 (0.77, 2.51) | 0.268 |
| BMI, kg/m^2^ | 24.97 ± 3.23 | 1.06 (0.98, 1.14) | 0.169 |
| **Vascular Risk Factors** | | | |
| Hypertension, *n* (%) | 198 (78.26%) | 1.48 (0.78, 2.81) | 0.226 |
| Diabetes mellitus, *n* (%) | 111 (43.87%) | 0.87 (0.52, 1.45) | 0.580 |
| Dyslipidemia, *n* (%) | 153 (60.47%) | 1.00 (0.59, 1.67) | 0.988 |
| Coronary heart disease, *n* (%) | 32 (12.65%) | 0.50 (0.22, 1.17) | 0.112 |
| Smoking status, *n* (%) | 79 (31.23%) | 1.36 (0.79, 2.35) | 0.261 |
| SBP, mmHg | 142.26 ± 19.39 | 1.01 (1.00, 1.02) | 0.130 |
| DBP, mmHg | 83.15 ± 12.21 | 1.00 (0.98, 1.02) | 0.789 |
| **Laboratory Findings** | | | |
| Fasting glucose, mmol/L | 6.60 ± 2.36 | 0.98 (0.88, 1.10) | 0.742 |
| HbA1c, % | 6.98 ± 1.66 | 1.07 (0.92, 1.25) | 0.385 |
| Total cholesterol, mmol/L | 4.35 ± 1.01 | 1.21 (0.94, 1.56) | 0.135 |
| LDL-C, mmol/L | 2.60 ± 1.19 | 1.67 (1.33, 2.10) | **<0.001** |
| HDL-C, mmol/L | 1.06 ± 0.30 | 0.37 (0.15, 0.89) | **0.026** |
| Triglycerides, mmol/L | 1.64 ± 0.89 | 1.04 (0.78, 1.39) | 0.774 |
| Homocysteine, μmol/L | 12.59 ± 6.30 | 0.99 (0.95, 1.04) | 0.782 |
| Lp(a), mg/L | 188.46 ± 93.90 | 1.00 (1.00, 1.00) | 0.200 |
| Lp-PLA2, ng/mL | 184.95 ± 53.96 | 1.00 (1.00, 1.01) | **0.055** |
| **Inflammatory & Coagulation Markers** | | | |
| hs-CRP, mg/L | 6.61 ± 5.25 | 1.33 (1.24, 1.44) | **<0.001** |
| Fibrinogen, g/L | 3.25 ± 1.10 | 1.67 (1.30, 2.13) | **<0.001** |
| D-dimer, mg/L | 1.00 ± 0.83 | 1.77 (1.29, 2.43) | **<0.001** |
| **Hemodynamic Parameter** | | | |
| VAVF, ml/min | 139.17 ± 52.19 | 0.98 (0.97, 0.99) | **<0.001** |
| **Medication History** | | | |
| Antiplatelet therapy, *n* (%) | 77 (30.43%) | 0.42 (0.23, 0.77) | **0.005** |
| Statin therapy, *n* (%) | 88 (34.78%) | 0.24 (0.13, 0.44) | **<0.001** |

**Abbreviations:** BMI, body mass index; CI, confidence interval; DBP, diastolic blood pressure; HbA1c, glycated hemoglobin; HDL-C, high-density lipoprotein cholesterol; hs-CRP, high-sensitivity C-reactive protein; LDL-C, low-density lipoprotein cholesterol; Lp(a), lipoprotein(a); Lp-PLA2, lipoprotein-associated phospholipase A2; OR, odds ratio; PCI, posterior circulation infarction; SBP, systolic blood pressure; TC, total cholesterol; TG, triglycerides; VAVF, vertebral artery volume flow. **Note:** Data are presented as mean ± SD or number (%). Univariate logistic regression was used to calculate unadjusted ORs.
