# Supplementary material for: Inflammatory-hemodynamic interplay in severe intracranial stenosis: hs-CRP, vertebral artery flow, and posterior circulation infarction
Source: Front Neurol. 2026 Aug 25;17:1829231. doi: 10.3389/fneur.2026.1829231 (PMC13549819; doi:10.3389/fneur.2026.1829231)
Supplement: Supplementary file 2 [file Table_2.DOCX]

**Supplementary Table 2. Sensitivity Analyses of the Association Between Baseline hs-CRP and Incident PCI**

| **Analysis** | **Model** | **Participants/events, n** | **Effect estimate (95% CI)** | **P value** |
| --- | --- | --- | --- | --- |
| Cox regression | I | 190 / 33 | HR, 1.28 (1.21-1.35) | <0.001 |
|  | II | 190 / 33 | HR, 1.28 (1.21-1.35) | <0.001 |
|  | III | 190 / 33 | HR, 1.37 (1.24-1.53) | <0.001 |
| Restricted logistic regression | I | 190 / 33 | OR, 1.41 (1.27-1.56) | <0.001 |
|  | II | 190 / 33 | OR, 1.42 (1.27-1.59) | <0.001 |
|  | III | 190 / 33 | OR, 1.56 (1.31-1.87) | <0.001 |

**Abbreviations:** CI, confidence interval; HR, hazard ratio; hs-CRP, high-sensitivity C-reactive protein; LDL-C, low-density lipoprotein cholesterol; OR, odds ratio; PCI, posterior circulation infarction; VAVF, vertebral artery volume flow. **Note:** Effect estimates are reported per 1-mg/L increase in hs-CRP. Cox regression models report HRs, whereas restricted logistic regression models report ORs. Model I was unadjusted. Model II was adjusted for age and sex. Model III was adjusted for age, sex, LDL-C, VAVF, and statin therapy. Both analyses included 190 patients, comprising 33 patients with available PCI onset dates and 157 patients who remained event-free; patients with PCI but unavailable event timing were excluded. No evidence of violation of the proportional hazards assumption was observed in the Cox models (all Schoenfeld P > 0.05).
